# Ethical and Practical Considerations for an Agreement to Ensure Equitable Vaccine Access: Comment on "More Pain, More Gain! The Delivery of COVID-19 Vaccines and the Pharmaceutical Industry’s Role in Widening the Access Gap"

**DOI:** 10.34172/ijhpm.2024.8516

**Published:** 2024-05-18

**Authors:** Gustavo Ortiz-Millán

**Affiliations:** Instituto de Investigaciones Filosóficas, Universidad Nacional Autónoma de México, Ciudad Universitaria, Mexico City, Mexico.

**Keywords:** COVAX, Vaccine Agreement, Equitable Vaccine Distribution, Pharmaceutical Companies, Voluntary Licensing Agreements, Pandemic Preparedness

## Abstract

This paper discusses the potential of an international agreement to ensure equitable vaccine distribution, addressing the failures witnessed during the COVID-19 pandemic. COVAX was unable to prevent vaccine monopolization and unequal distribution, which led to significant disparities in vaccination rates and avoidable deaths. Any future agreement on equitable vaccine distribution must address ethical and practical issues to ensure global health equity and access. The proposed agreement should recognize healthcare as a human right and consider vaccines beyond mere commodities, emphasizing the social responsibility of pharmaceutical companies to prioritize affordability, availability, and accessibility, particularly for low-income countries (LICs). Voluntary licensing agreements are suggested as a means to enhance access to essential medicines. The paper also outlines the necessity of international cooperation, with robust compliance mechanisms, to effectively enforce such an agreement and mitigate future health crises.

## The Need of an International Agreement on Vaccine Distribution

 In April 2020, anticipating vaccine monopolization by high-income countries (HICs), global health organizations established COVAX for equitable vaccine access. By the end of 2020, 190 nations joined COVAX, yet challenges, including supply constraints, distribution issues, and financial constraints hindered its effectiveness. Pharmaceutical companies committed to equitable distribution, but ended up prioritizing HICs, pricing vaccines higher for low-income countries (LICs).^1^ Meanwhile, HICs decided to prioritize the procurement of vaccines for their own populations, incurring in what has been characterized as vaccine nationalism, which exacerbated global inequalities.^2^ By 2021, while HICs achieved a coverage rate of around 75%, only 2% of LICs were vaccinated, resulting in avoidable deaths.^3^ By April 2024 (four months after the end of COVAX, on December 31, 2023), inequalities remain, 70.6% of the world population has received at least one dose of a COVID-19 vaccine, but only 32.7% of people in LICs have received at least one dose.^4^ Lingering disparities highlight the failure to fulfill commitments and mitigate the pandemic’s toll on vulnerable populations.

 Borges et al^5^ want that an unequal distribution such as the one witnessed during the COVID-19 pandemic does not happen again. They claim that “there is a need to approve an international treaty that targets the activities of all actors, including the pharmaceutical companies, in protecting human rights and the right to health realms.”^4^ Such a treaty should not only involve pharmaceutical companies, but also governments, international organizations, and civil society. It is a good initiative, but for it to work out, some ethical and practical considerations should be taken into account. Even though they present the idea of a general treaty that “regulates the activities of pharmaceutical companies in the area of human rights,” which is very ambitious, I am going to consider some of the ethical and practical issues of a slightly less ambitious treaty, one which builds on the COVAX experience and addresses the issue of an equitable global vaccine distribution in case of another pandemic. Maybe some of the considerations for such a treaty could be useful for a more general treaty, like the one proposed by Borges et al—or for the agreement on pandemic preparedness the World Health Organization (WHO) is working on.^6^

## Ethical Considerations

 An international agreement on equitable vaccine distribution should ideally recognize a universal right to healthcare. Access to healthcare, including vaccines, is essential for individuals to live a life of dignity. Recognizing healthcare as a human right acknowledges the inherent value and worth of every individual, regardless of their socio-economic status or nationality. By treating healthcare as a human right, governments and international organizations prioritize policies and actions that promote health and prevent disease, including the equitable distribution of vaccines. Ensuring universal access to healthcare—of which vaccines are a crucial component—promotes social justice by addressing disparities in health outcomes. Vulnerable and marginalized populations, but also LICs, who often face barriers to healthcare access, benefit from policies that recognize healthcare as a fundamental human right.

 At the same time, any such treaty should acknowledge that vaccines transcend mere commodities; how they are conceived and the approaches to vaccine distribution should be discussed (for instance, Emanuel and colleagues have distinguished four approaches: tiered pricing, global public goods, partly bilateral, and fully multilateral approaches^7^). Whether created by private or public-private entities, vaccines offer benefits that extend beyond borders and markets. Consequently, they should not be perceived solely as commodities susceptible to privatization.

 Similarly, pharmaceutical companies should not be viewed merely as economic entities bound exclusively by legal and contractual obligations. This limited perspective implies that they would only address societal issues if doing so is profitable, which undermines their broader social responsibilities. The moral obligations of these companies during a pandemic should be discussed. It should be considered that they have a responsibility to ensure equitable access to essential medicines, including vaccines and treatments for a pandemic. This involves prioritizing affordability, availability, and accessibility, particularly for vulnerable populations and LICs. They also should uphold principles of transparency and accountability in their operations, including pricing, licensing agreements, and regulatory interactions. This includes disclosing information about research findings, manufacturing processes, and financial transactions related to pandemic response efforts. Part of their social responsibility is to adhere to regulatory standards and guidelines in the fair distribution of vaccines in the context of a pandemic. Pharmaceutical companies are collective citizens with responsibilities towards global health. With this view in mind, and realizing that they have an enormous political power, they may be able to contain the vaccine nationalism of individual national governments.

 Voluntary licensing agreements should also be considered, since they could be seen as a manifestation of corporate social responsibility. These agreements, where patent holders allow others to produce their drug formulations or vaccines, could greatly enhance access to essential medicines for vulnerable populations and LICs, which is vital during a global health crisis.^8^ Strategies to facilitate such agreements could involve creating frameworks that incentivize pharmaceutical companies to engage in voluntary licensing. This could include tax benefits, public recognition, or expedited review processes for other products from companies that contribute to such initiatives. International bodies and agreements, like the proposed agreement, could lay down guidelines for voluntary licensing to ensure that it becomes a standard practice during pandemics. Transparency in licensing terms, ensuring fair pricing, and access clauses could be mandated to align private interests with the global public good.^9^

 COVAX was rightly criticized from an ethical point of view. It would be beneficial to define what is meant by “equity” in this context and how it relates to vaccine distribution. Critics rightfully pointed out shortcomings in COVAX’s distribution policy, particularly the fact that its distribution approach primarily focused on proportionality: distributing vaccines equally based on each country’s population size. This approach assumes that fairness entails treating all countries the same, rather than addressing their unique circumstances and requirements equitably. Varying needs among countries should be adequately considered.^10^

## Practical Challenges

 The aforementioned ethical points are among the foundational aspects on which a treaty or agreement on equitable global vaccine distribution should be based, but there are practical aspects and challenges that should also be considered.

 (1) The first one is that countries may view vaccine-related decisions as matters of national sovereignty, making it difficult to enforce an international treaty that dictates vaccine distribution or mandates. In a time of populist nationalism, when governments and many people are suspicious of constraints coming from supranational organizations and international treaties, which are seen as constraining the “will of the people,” this may be something hard to achieve.^11^ The institutions in charge of implementing the treaty should try to work with national governments to show them the advantages of participating in such an agreement.

 (2) The unequal global distribution of vaccines during the COVID-19 pandemic presented a classic problem of collective action: the pursuit of self-interest by pharmaceutical companies, coupled with the self-interest of individual HICs, failed to yield an effective and equitable allocation of vaccines.^12^ The proposed agreement should create incentives that align individual self-interest with collective goals. For example, governments and international agencies could offer rewards or subsidies to companies that prioritize equitable vaccine distribution, thereby encouraging cooperation for the greater good. The agreement should also include mandating transparency in vaccine distribution processes, penalizing companies or countries that prioritize profit over equity. If there are no clear sanctions for both individual governments and pharmaceutical companies that fail to comply with their commitments, the individual interest of each of these may prevail; especially if failing to comply can bring greater economic or political benefits than complying.

 In the context of the discussion about the proposed WHO pandemic preparedness agreement, Kavanagh et al have suggested several specific mechanisms that, together, could create compliance pressures to shift state behavior: Establishing a Conference of the Parties as a global governance mechanism with oversight of compliance could prove pivotal. It could embody a policing function, bolstered by independent rapporteurs conducting investigatory missions and delivering thematic reports. These rapporteurs would address complaints from both states and individuals. Moreover, formal dispute settlement mechanisms, accessible to individual countries, could offer avenues for resolution while potentially deterring non-compliance through soft retaliation (eg, withdrawing cooperation or benefits within the agreement). Instituting a structured framework for civil society involvement, beyond mere observer status, could involve shadow reporting from academic and civil society entities. Additionally, a platform for assistance requests would allow compliant yet resource-limited countries to seek technical or financial aid. Lastly, embedding trust-building activities into law would ensure consistent practices linked directly to fostering trust and compliance.^13^

 (3) The treaty should also establish international oversight mechanisms, and foster international cooperation and collaboration to address global challenges collectively. Strengthening institutions like the WHO, the Coalition for Epidemic Preparedness Innovations, the Global Alliance for Vaccines and Immunizations, the International Coalition of Medicines Regulatory Authorities, or even the World Trade Organization, can promote compliance of the agreement.

 (4) The financing of equitable vaccine distribution in a pandemic scenario, particularly for countries facing debt distress and competing priorities, presents a significant challenge. To address this, the global community can consider several mechanisms to ensure that financial constraints do not impede access to vaccines. These are some of the mechanisms that may be considered: (*i*) Global solidarity funds: Establishing a dedicated fund for pandemic response, to which countries contribute based on their economic capabilities, could be one way to finance vaccine distribution. HICs would contribute more, while LICs would contribute less or receive exemptions.^14^ (*ii*) Debt relief initiatives: International financial institutions could offer debt relief or restructuring to LICs, freeing up resources for health spending.^15^ (*iii*) Tiered contributions: Following the principle of common but differentiated responsibilities, countries could contribute to a global vaccine fund based on a tiered system that considers their gross domestic product, healthcare spending, and existing healthcare capabilities. Inclusion of the concept of common but differentiated responsibilities in a vaccine distribution agreement may acknowledge that while all countries share the responsibility of global health security, they do not all have the same capabilities to contribute financially or technically. This principle can ensure that the burden of pandemic response does not fall disproportionately on countries that are less able to bear it, fostering a more equitable global response.^16^ (*iv*) Public-private partnerships: Encourage partnerships between governments and private sector entities to finance vaccine production and distribution, leveraging corporate social responsibility initiatives.^17^ (*v*) Innovative financing instruments: Implement financial instruments like pandemic bonds, vaccine bonds, or social impact bonds, which can raise funds from the capital markets for pandemic preparedness and response.^18^ (*vi*) Multilateral development banks: Leverage the capacity of these banks to provide low-interest loans or grants specifically for pandemic preparedness, including the development of healthcare infrastructure.^19^ (*vii*) Philanthropic contributions: Foundations and charities can play a role by providing grants for vaccine procurement and distribution, especially in regions that are most vulnerable. (*viii*) International donor coordination: Ensure that donor funding is well-coordinated to avoid duplication of efforts and to channel funds to where they are most needed, according to an agreed-upon international framework.

 (5) The agreement should consider investments in long-term planning and infrastructure development, technical aspects and support for readiness before the arrival of vaccines in LICs. For instance, it should promote investment in cold chain infrastructure and other system aspects. This helps to build capacity that may be useful for future health emergencies as well as for other healthcare and vaccination campaigns. COVAX considered these investments, but in some cases they did not come fast enough.^20^ It should also consider investing in getting the necessary human resources in place and training them and strengthening social safety nets, all of which may be crucial for an equitable vaccine distribution. International organizations could take a lead role in incentivizing countries to bring a diversity of stakeholders together.

 (6) The epidemiological landscape is dynamic, with distinct phases in different countries where different health measures must be taken, and where the distribution of vaccines has to be sensitive to these variations. Also new diseases and variants may emerge over time, as was the case with COVID-19. Enforcing an agreement that addresses evolving health threats, regional epidemics, and pandemics requires adaptability and flexibility.

 (7) The agreement should address the need to scale manufacturing capacity. Depending on a small number of HICs to donate millions of doses may not work as well as having some low- and middle-income countries manufacturing the vaccines themselves, particularly across Africa, which in 2021 imported 99% of its vaccines while lacking the pre‐order purchasing capacity of HICs.^21^ Enforcing an agreement that mandates an equitable vaccine distribution may be challenging without addressing disparities in manufacturing capacity. Diversifying manufacturing capacity can mitigate supply chain disruptions, reduce dependency on external sources, and improve access to life-saving vaccines during health emergencies.

 Any agreement or treaty such as the one I have been commenting (the one proposed by Borges et al, but also the one the WHO is working on) would have to examine the other existing global health agreements to identify potential areas of collaboration. Rouw et al identified 71 agreements as having a role in global health; about a third (21 or 30%) of these have pandemic preparedness and response as part of their original mandate, and most of these agreements (50 or 70%) participated in the COVID-19 response.^22^

 Finally, even if an agreement regulating vaccine distribution may seem to be necessary, we should take into account that recent research tells us that treaties have often fallen short of achieving their intended outcomes—except in the realms of international trade and finance. Hoffman et al suggest that impactful treaties succeed through socialization and normative processes rather than relying solely on long-term legal mechanisms. Moreover, the inclusion of enforcement mechanisms is the key adjustable factor that could enhance the effectiveness of treaties governing various policy domains, including healthcare issues. This casts doubt on the efficacy of international treaties that fail to incorporate enforcement mechanisms.^23^ Addressing these challenges requires a coordinated and collaborative approach among governments, international organizations, civil society, and the private sector. While enforcing an international treaty on equitable global vaccine distribution presents difficulties, it remains essential for ensuring a fair access to vaccines and combating future global health threats effectively. That an unequal distribution such as the one witnessed during the COVID-19 pandemic does not happen again is in the interest of everybody.

## Acknowledgements

 The author acknowledges the support of the Oxford Uehiro Centre for Practical Ethics, University of Oxford, where this paper was written.

## Ethical issues

 Not applicable.

## Competing interests

 Author declares that he has no competing interests.

## Funding

 This work was supported by the Dirección General de Asuntos del Personal Académico of the Universidad Nacional Autónoma de México, through a Programa de Apoyos para la Superación del Personal Académico (PASPA) grant.
